# Immune transcriptome analysis of COVID-19 patients infected with SARS-CoV-2 variants carrying the E484K escape mutation identifies a distinct gene module

**DOI:** 10.1038/s41598-022-06752-0

**Published:** 2022-02-18

**Authors:** Hye Kyung Lee, Ludwig Knabl, Ludwig Knabl, Manuel Wieser, Anna Mur, August Zabernigg, Jana Schumacher, Sebastian Kapferer, Norbert Kaiser, Priscilla A. Furth, Lothar Hennighausen

**Affiliations:** 1 National Institute of Diabetes, Digestive and Kidney Diseases, Bethesda, MD 20892 USA; 2 TyrolPath, Zams, Austria; 3 Krankenhaus St. Vinzenz, Zams, Austria; 4 Division of Internal Medicine, Krankenhaus Kufstein, Kufstein, Austria; 5 Division of Internal Medicine, Krankenhaus St. Johann, St. Johann, Austria; 6 Departments of Oncology and Medicine, Georgetown University, Washington, DC USA

**Keywords:** Computational biology and bioinformatics, Diseases, Medical research, Gene expression

## Abstract

Fast-spreading variants of severe acute respiratory syndrome coronavirus 2 (SARS-CoV-2) energize the COVID-19 pandemic. While viral infections elicit a conserved immune response, it is not known whether SARS-CoV-2 variants, which display enhanced binding to the ACE2 receptor and reduced neutralizing activity by vaccine-elicited antibodies, prompt specific genomic immune responses. To test this, we generated and investigated the transcriptomes in BCs from hospitalized patients infected with either the Alpha variant (n = 36) or with the Alpha variant that had acquired the E484K escape mutation (Alpha+E484K) (n = 13). We identified a gene module preferentially activated in patients infected with the Alpha+E484K variant and in patients infected with the Beta (n = 9) and Gamma (n = 3) variants that also carry by the E484K escape mutation. The E484K signature was enriched for genes preferentially expressed in monocytes and linked to severe viral infection. However, both cohorts had undergone similar treatments and no differences in disease severity were reported suggesting that this signature reflects a variant response and does not necessarily associate with disease outcome. Additionally, longitudinal transcriptome analyses revealed a more persistent retention of immune signatures in Alpha+E484K patients throughout the entire course of COVID-19 disease and convalescence. While the OAS1 Neanderthal mutation has been linked to a milder COVID-19 pathology, we did not identify significant immune transcriptomes differences in the 25 patients homozygous for this mutation. Our study offers insights into distinct molecular immune responses elicited by SARS-CoV-2 variants carrying the E484K escape mutation throughout the COVID-19 disease.

## Introduction

The cytokine storm triggered by SARS-CoV-2 infection invariably influences the peripheral immune cells leading to systemic host responses. Indeed, RNA-seq studies using whole blood or peripheral blood mononuclear cell (PBMC) samples have provided extensive knowledge of the transcriptomes of immune cells from COVID-19 patients^1–7^. In general, genetic pathways under the control of interferons and JAK-STAT signaling are activated up to several 100-fold in COVID-19 patients.

Variants of concern (VOC) carrying specific mutations thought to enhance viral fitness have emerged and continue to emerge. The Beta (formerly B.1.351)^8^ and Gamma (formerly P.1) variants were of particular concern because they carry the mutation E484K within the receptor binding domain (RBD). E484 is an immunodominant spike protein residue and the E484K substitution enables the escape from neutralizing antibody inhibition in vitro^9–11^ and it may be linked with reduced vaccination efficacy^12^.

In February 2021 Public Health England (PHE) published a report of Alpha (formerly B.1.1.7) genomes with acquisition of the E484K spike mutation^13^. Notably, the presence of the E484K mutation into the Alpha background led to a more-substantial loss of neutralizing activity by vaccine-elicited antibodies compared with the mutations in Alpha alone^9,10^, suggesting that this variant represents a threat to the efficacy of the BNT162b2 mRNA vaccine.

Published studies on the immune transcriptomes of COVID-19 patients have not specified the underlying SARS-CoV-2 variants. Thus, it remains unclear whether variants can elicit specific and unique molecular immune response. To address this question, we investigated the immune transcriptomes in hospitalized patients infected with the Alpha+E484K variant or the parent Alpha variant. This study tested the null hypothesis that infection with the different variants would not alter the immune host transcriptional profile. The data revealed a stronger immediate immune response of patients infected with the Alpha+E484K variant as compared to the parent Alpha variant. Using RNA-seq, a variant-agnostic approach, we identified conserved and unique host responses to E484K variants, including Beta and Gamma.

## Results

### Immune transcriptome responses of hospitalized patients infected with the SARS-CoV-2 Alpha and Alpha+E484K variants

**Figure 1:**
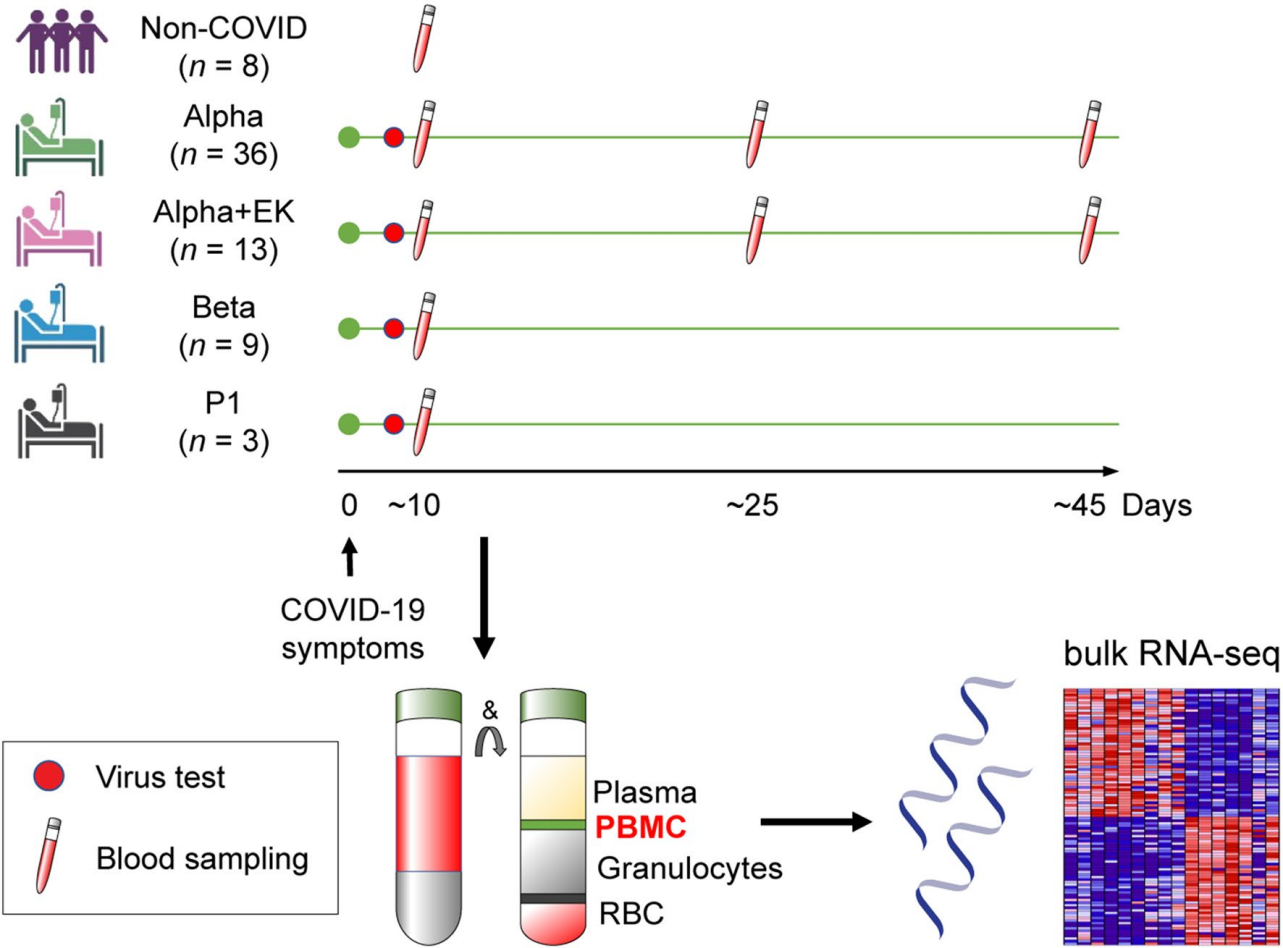
Experimental design. Schematic presentation of the patient cohort and the experimental workflow. COVID-19 patients were diagnosed, and the variants were identified through whole viral genome sequencing. Buffy Coast (BC) cells including PBMCs and granulocytes were purified from blood drawn at different times after symptomology and RNA-seq was performed at a depth of 215 million reads per sample.

While there is extensive information on genetic pathways activated in immune cells of hospitalized COVID-19 patients, the impact of different variants, in particular those carrying the E484K escape mutation, is not clear. Here, we investigated the bearing of the E484K escape mutation on the immune response and analyzed the Buffy Coat (BC) transcriptomes of patients infected with the SARS-CoV-2 Alpha variant and the Alpha variant carrying the E484K escape mutation. A total of 36 hospitalized patients infected with the Alpha variant and 13 patients infected with the Alpha+E484K variant participated in this study (for detailed information see Table 1 and Supplementary Table 1). Blood samples were collected during three distinct time windows following first symptomology (Fig. 1), during hospitalization, after discharge and during convalescence (Supplementary Table 1). RNA-seq was conducted on a total of 100 samples. As non-COVID controls, we conducted RNA-seq on BCs collected from eight healthy, naïve individuals that were part of a BNT162b2 vaccination study.

**Table 1:** Demographic and Clinical Characteristics of COVID-19 patients infected by the Alpha or Alpha+E484K SARS-CoV-2 variants.

Type of SARS-CoV-2 virus	Alpha	Alpha + EK	Significant	Chi-square	*p* value
Patients, *n* (%)	36	13			
Patient age (mean)	71	67	No	0.56	0.91
> 65 yr, *n* (%)	25 (69.4)	8 (61.5)			
41–65, *n* (%)	8 (22.2)	3 (23.1)			
21–40, *n* (%)	3 (8.3)	2 (15.4)			
Gender			No	0.51	0.77
Females, *n* (%)	18 (50.0)	5 (38.5)			
Males, *n* (%)	18 (50.0)	8 (61.5)			
BMI (mean)	25	24.3	No	0.87	0.93
Underweight (< 18.5), *n* (%)	1 (2.8)	1 (7.7)			
Normal (18.5–24.9), *n* (%)	12 (33.3)	5 (38.5)			
Overweight (25.0–29.9), *n* (%)	8 (22.2)	4 (30.8)			
Obese (≧30.0), *n* (%)	4 (11.1)	1 (7.7)			
Vaccination, *n* (%)	9 (25.0)	4 (30.8)	No	0.09	0.30
Treatment, *n* (%)	24 (66.7)	8 (58.3)	No	0.72	0.87
Corticosteroid	23 (64.9)	8 (58.3)			
Convalescent plasma	2 (5.6)	0			
ICU, *n* (%)	7 (19.4)	3 (23.1)	No	0.05	0.82
Deaths, *n* (%)	6 (16.7)	4 (30.7)	No	0.73	0.39
Data set	67	30			
During hospitalization, *n* (%)	32 (88.9)	13 (100)	No	0.07	0.80
After discharge, *n* (%)	30 (83.3)	10 (77.0)	No	0.03	0.87
During convalescence, *n* (%)	5 (13.9)	7 (53.8)	Yes	4.42	0.04

To address whether or not any additional polymorphisms in the viral genome tracked with the E484K mutation we examined the full viral sequences from all individuals for which the data was available (Supplementary Table 2). We then examined these polymorphisms to determine if they resulted in amino acid changes. We did not identify any additional amino acid mutations specific to the Alpha+E484K variant. A C > T transition at nt 22,000 did not result in an amino acid change.

Initially, we investigated the immune transcriptomes of patients infected with either the Alpha+E484K or the Alpha parent variant within 10 days after the onset of COVID-19 symptomology. All patients had been hospitalized and there were no significant differences in age distribution, gender, BMI, vaccination status, treatment, ICU and deaths between the two groups (Table 1). To gauge the possibility of changes in cell populations between the two experimental groups we conducted complete blood count (CBC) analyses (Supplementary Fig. 1). No significant differences were detected in BC components in the two populations (Supplementary Fig. 1) and the time since symptom onset (Supplementary Fig. 2).

**Figure 2:**
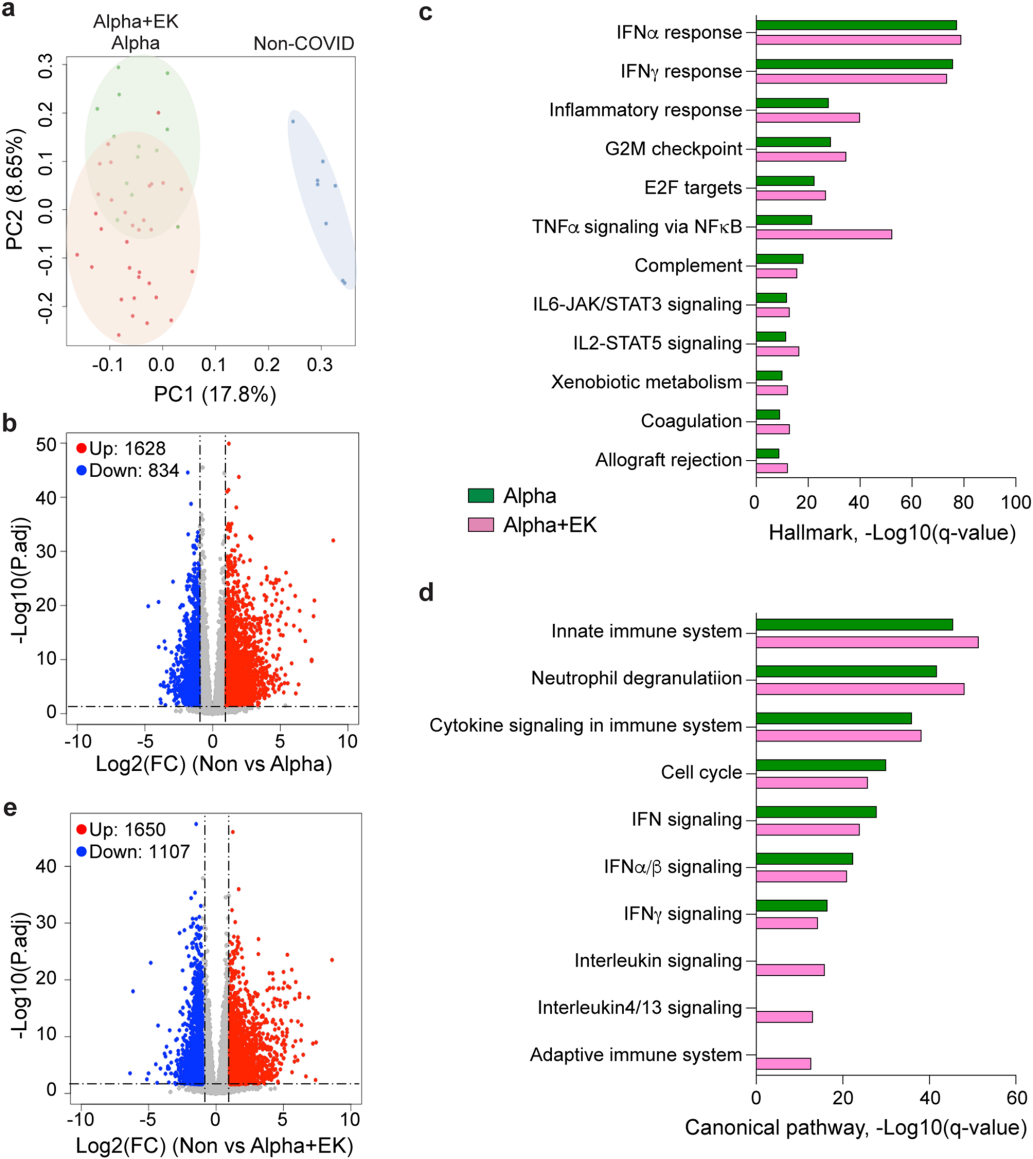
Immune transcriptomes in patients infected with the Alpha or Alpha + EK variants. (**a**) Principal-component analysis (PCA) of transcriptomes of 35 hospitalized patients infected with the Alpha variant (red) and 13 infected with the Alpha + EK variant (green) within 10 days of first symptomology. Eight healthy non-COVID (blue) served as controls. The PCA depicts the variation in the global gene expression profiles across the three cohorts. Principal components 1 (PC1) and 2 (PC2), which represent the greatest variation in gene expression, are shown. (**b**) Volcano plot of DEGs comparing Non-COVID versus Alpha. DEGs (adjusted *p*-value, *p*. adj < 0.05) with a log2 fold change (FC) of more than 1 or less than − 1 are indicated in red and blue, respectively. Non-significant DEGs are indicated in gray. The numbers of upregulated and downregulated genes are listed. (**c**, **d**) Genes expressed at significantly higher levels in the COVID-19 patients were significantly enriched in Hallmark Gene Sets (**c**) and Canonical Pathway Gene sets (**d**). X-axis denotes statistical significance as measured by minus logarithm of FDR q-values. Y-axis ranked the terms by q values (green bars, Alpha; pink, Alpah + EK). (**e**) Volcano plot of DEGs comparing Non-COVID versus Alpha + EK.

BCs were isolated from the 36 hospitalized Alpha patients and 13 hospitalized Alpha+E484K patients and RNA-seq was conducted with an average of 215 million reads per sample passing quality control. PCA plot showed separation between RNA-seq samples from non-COVID healthy controls and the two variants on first and second principal components (PC1 and PC2) (Fig. 2a). A total of 2,462 differentially regulated genes (DEGs) were obtained in patients infected with Alpha as compared to the healthy controls (Fig. 2b; Supplementary Table 3). Expression of 1628 genes was more than twofold induced and expression of 834 genes was reduced at least two-fold. As expected, and as shown before, these genes were highly enriched for interferon-regulated pathways, innate immune response and the JAK-STAT signaling pathway (Fig. 2c–d). In the Alpha+EK patients a total of 2757 genes were differentially expressed as compared to the healthy controls with 1650 being induced at least two-fold (Fig. 2e; Supplementary Table 4). The enrichment for immune-regulated pathways was equivalent for both variants (Fig. 2c–d).

**Figure 3:**
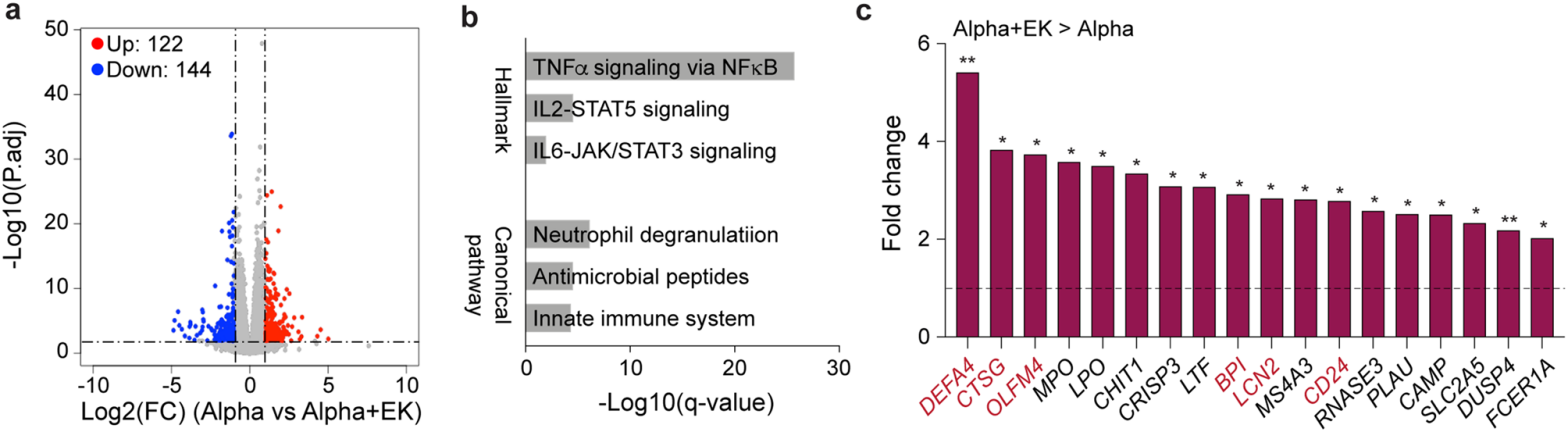
Differences of immune response between hospitalized patients infected with the Alpha or Alpha + EK variant. (**a**) The volcano plot shows the DEGs between the Alpha and Alpha + EK patients. The numbers of upregulated and downregulated genes are listed. (**b**) Hallmark and Canonical pathway analysis of significantly enriched genes included in clusters for innate immune-related biological processes. (**c**) Relative mRNA levels of 18 immune associated genes that are induced in the Alpha + EK patients as compared to the Alpha patients and enriched in innate immune response are presented by bar graphs. The genes of neutrophil degranulation are shown in red. DESeq2 *p*-adj from wald test using Benjamini and Hochberg method are listed in Supplementary Table 4. *p < 0.05, **p < 0.001.

A direct comparison of RNA-seq data from patients infected with the Alpha or Alpha+E484K variant revealed the differential expression of 266 genes, with 122 being induced in the Alpha+E484K patients by at least two-fold (Fig. 3a; Supplementary Table 5). These genes group in distinct pathways, including TNFα and JAK-STAT signaling have been shown to control neutrophil biology (Fig. 3b). Several genes in this group (*CTSG*, encoding the neutrophil serin protease Cathepsin G; *DEAFA4*, defensin alpha 4; *LCN2*, lipocalin 2; *OLFM4*, *BPI* and *CD24*) (Fig. 3c), are known to be overexpressed in patients with severe viral infections, including COVID-19^7,14^. However, since there were no significant differences in clinical severity between the Alpha and Alpha+E484K groups reported (Supplementary Table 1), these genes might be part of a module particularly sensitive to SARS-CoV-2 E484K variants. No pathway assignment was obtained for genes whose expression was reduced in Alpha+E484K patients (Supplementary Table 5).

Six out of the 36 patients infected with the Alpha variant and 4 out of the 13 patients infected with the Alpha+E484K variant died within 2–4 weeks after first symptomology (Supplementary Table 1). A direct comparison of gene expression between these two groups early into their hospitalization revealed a limited number of DEGs that provide clues about the disease progression (Supplementary Table 6). Among them are *DEFA1* (an antiviral protein expressed in neutrophils), ORM1 and OLAH, a metabolic protein involved in fatty acid synthesis.

### Gene module linked to the E484K escape mutation

**Figure 4:**
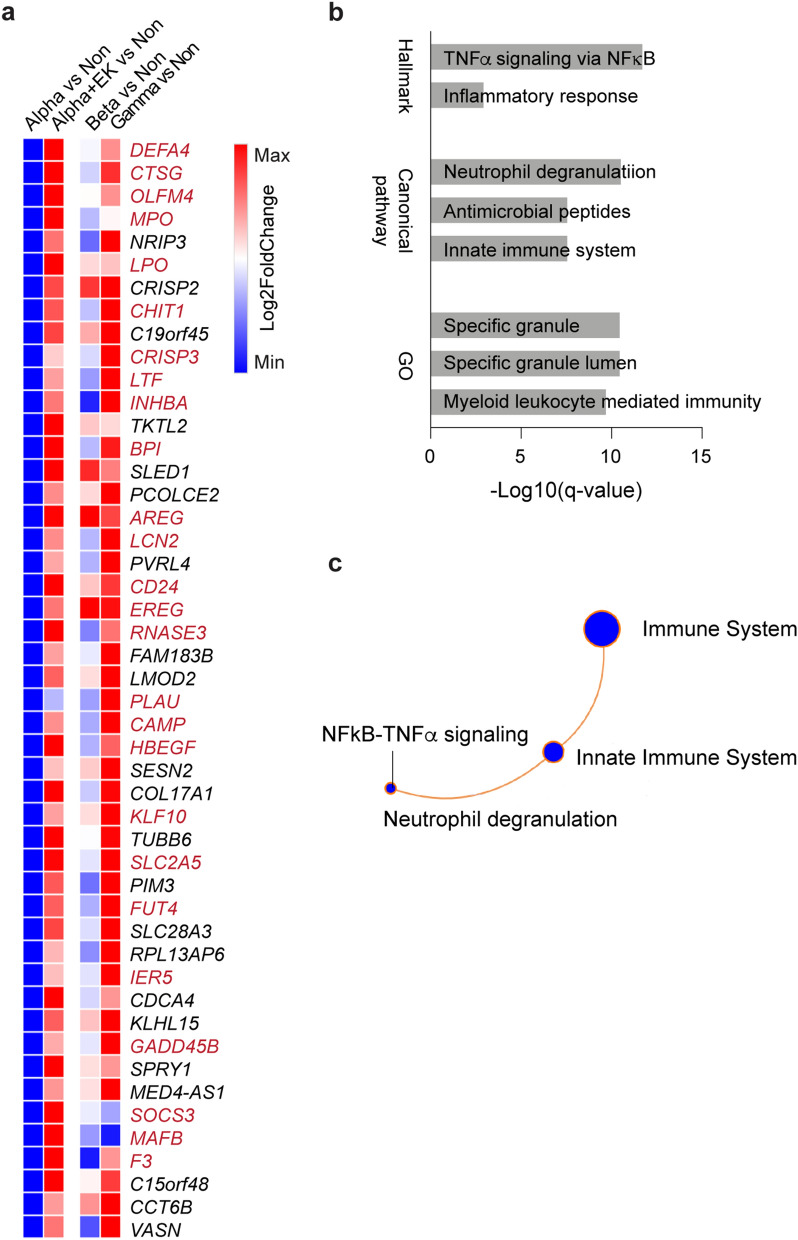
Distinct immune transcriptome signature in COVID-19 patients infected by different SARS-CoV-2 variants carrying the E484K mutation. (**a**) Heatmap shows the expression levels of 48 genes significantly induced by variants with the E484K mutation as compared to Non-COVID controls. Immune-related genes are in red. (**b**, **c**) GSEA analysis for Hallmark, Canonical pathway and Gene ontology (GO) of the 48 genes shows the enrichment of neutrophil degranulation via NFκB-TNFα signaling.

Our study demonstrated that the acquisition of the E484K escape mutation in the Alpha background is associated with a heightened expression of a subset of immune-associated genes suggesting a distinct host response to the E484K variant. This finding begged the question whether this E484K module was specific to patients infected with the Alpha variant or also present in patients infected with other variants carrying the E484K mutation, such as Beta (formerly B.1.351) and Gamma (formerly P.1). For this, we mined immune transcriptomes we had generated from nine hospitalized patients infected with the Beta variant^15^. Out of the 122 genes overexpressed in the Alpha+E484K patients, 67 were also overexpressed in Beta patients (Supplementary Table 7). For this study we had also recruited three hospitalized patients infected with the Gamma variant and a total of 48 genes were significantly higher expressed in all three E484K variants (Fig. 4a, Supplementary Table 7). This 48 gene module is enriched for genes controlling neutrophil biology with NFκB-TNFα signaling (Fig. 4b–c). Of note, hospitalized patients infected with the four different variants received equivalent medical treatment, including Dexamethasone regimen (Supplementary Table 1), and there were no significant differences in clinical severity between any of the groups reported (Supplementary Table 1).

### Longitudinal analysis of the immune transcriptomes

**Figure 5:**
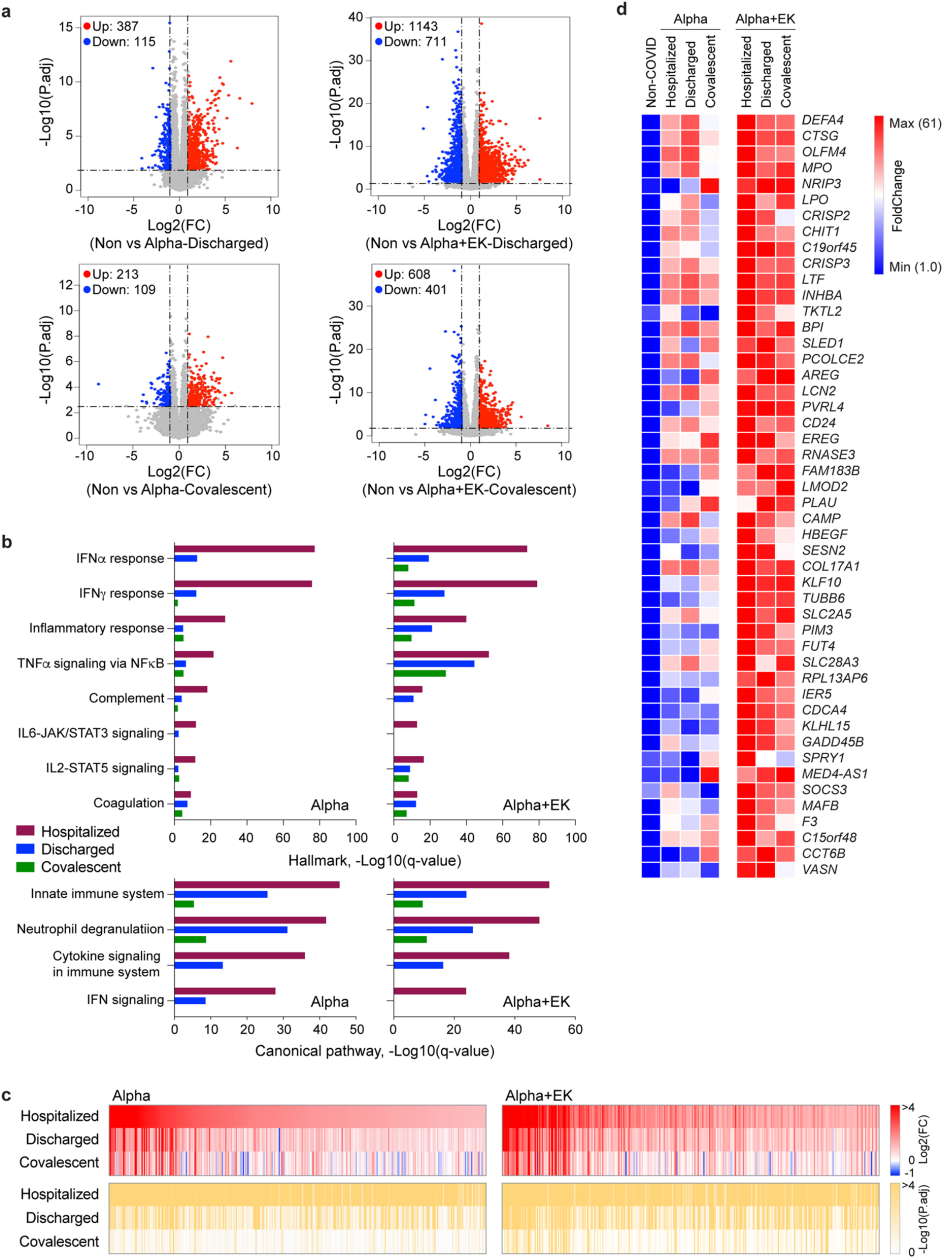
Longitudinal progression of immune transcriptomes from patients infected with the Alpha or Alpha + EK variants. (**a**) Volcano plot of DEGs comparing Non-COVID versus discharged or convalescent of the Alpha and Alpha + EK patients. The numbers of upregulated and downregulated genes are listed. (**b**) Genes expressed at significantly higher levels in the COVID-19 patients were significantly enriched in Hallmark Gene Sets (top) and Canonical Pathway Gene sets (bottom). X-axis denotes statistical significance as measured by minus logarithm of FDR q-values. Y-axis ranked the terms by q values (red bars: Hospitalized, blue bars, Discharged; green, Convalescent). (**c**) Heatmaps showing log2 FC (top) and corresponding P.adj (bottom) of gene sets from (**b**). (**d**) Heatmaps showing relative fold change of 48 signature genes for the E484K mutation.

While hospitalized patients infected with the Alpha+E484K variant displayed a more expansive immune transcriptome than the Alpha parent variant, it was not known whether the variants influenced the longitudinal progression of immune transcriptomes. To obtain a more nuanced view of the gene expression kinetics during disease progression and convalescence, we analyzed the temporal expression pattern of specific gene classes. We specifically addressed whether patients infected with the Alpha+E484K variant would retain a gene expression signature distinct from patients infected with the parent Alpha variant. For this, we directly compared the transcriptomes from the Alpha and the Alpha+E484K cohorts at approximately 25 days after first symptomology and again after an additional three weeks during convalescence (Fig. 5a). First, we generated transcriptomes from 28 Alpha patients and 10 Alpha+E484K patients after their discharge from the hospital, on average 25 days after first symptomology (Supplementary Tables 8, 9). Expression of a total of 387 genes was significantly induced in Alpha patients and 1143 genes were upregulated in Alpha+E484K patients (Fig. 5a). Next, we investigated the immune transcriptome in five Alpha patients and seven Alpha+E484K during convalescence, on average 45 days post first symptomology (Fig. 5a). While 213 genes with an elevated expression were detected in patients infected with the Alpha variant, 608 genes were still upregulated in the Alpha+EK patients (Fig. 5a; Supplementary Tables 10, 11). GSEA analyses demonstrated the preferential retention of immune-regulated gene signatures in patients infected with the Alpha + EK variant as far as nine weeks post first symptomology (Fig. 5b–c).

Specifically, we investigated the temporal fate of the 48 gene E484K module in the Alpha and Alpha+E484K cohorts (Fig. 5d; Supplementary Table 12). On average, in the Alpha cohort, the expression of this gene module declined 30% between T1 and T2 and 73% between T1 and T3. In contrasts, the decline was 2% between T1 and T2 and 10% between T1 and T3 in the Alpha+E484K cohort. These findings suggest that the E484K mutation elicits a more prolonged activation of immune pathways.

### Prevalence and impact of the OAS1 Neanderthal mutation

A study has shown that a major genetic risk factor for severe COVID-19 is linked to the Neanderthal genome^16^. A single point mutation in the *OAS1* gene affecting splicing, and possibly OAS1 levels, has been identified^17–19^. To follow up on these studies, we investigated whether patients carrying this mutation had a different disease outcome and possibly a unique transcriptome signature. A total of 24 patients (18 Alpha patients and 6 Alpha+E484K patients) were homozygous for the OAS1 mutation (Supplementary Table 13). No differences in the disease severity (Supplementary Table 1) and the immune transcriptomes were detected.

## Discussion

The COVID-19 pandemic and the emergence of new SARS-CoV-2 variants has highlighted the unmet need to understand variant-specific host immune responses. Here, we tested whether different variants elicit distinct genomic immunological responses in hospitalized COVID-19 patients. Through RNA-seq, a gene-agnostic approach, we identified a module of 48 genes expressed at distinctly higher levels in patients infected with variants carrying the E484K escape mutation as compared to the Alpha variant. Since the treatment of the different patient cohorts was equivalent, including Dexamethasone medication, and no differences in disease severity was reported, the pathological significance of this signature remains to be understood.

While variants are characterized by the acquisition of several mutations, our conclusions are, first and foremost, based on the native Alpha variant and an Alpha variant that had acquired the single point mutation resulting in the E484K escape variant. The Alpha lineage is defined by the presence of 23 nucleotide mutations across the genome, 14 of cause amino acid changes. Using the MetaSignature database (https://metasignature.stanford.edu), we determined that the majority of the genes in the E484K module are preferentially expressed in myeloid cells. Several of these genes (CAMP, LCN2) are known to have pro-inflammatory functions in myeloid cells and they have been linked to disease severity^14^ and strongly associate with COVID-19 critical illness and mortality^20^. Elevated neutrophil activation (LCN2) was also observed in post-acute sequelae of SARS-CoV-2 infection^21^. In another study, elevated expression of pro-neutrophil genes, including CD24, OLFM4, LCN2, and BPI, has been associated with poor outcome in sepsis^22^. However, the E484K signature is specific and does not merely reflect disease severity as other functional markers^20^, such as ARG1, ORM1, OLR1, LOX-1, CEACAM-8, MDSCs are not part of the signature.

Biological understanding of potentially distinct immune responses to SARS-CoV-2 variants was incomplete as previous studies had failed to identify the nature of the variants^1–7,23^. By controlling for the biological and clinical heterogeneity across data, we provide evidence of both a conserved host immune response to acute viral infection, irrespective of the SARS-CoV-2 variant, and unique responses to variants carrying the E484K escape mutation. RBD residue E484 has emerged repeatedly in the global SARS-CoV-2 population and is of principal importance as amino acid changes to K, Q or P reduce neutralization titers by more than an order of magnitude^24,25^. While the molecular understanding underlying the ability of the E484K mutation to contribute to a specific immune signature is incomplete, a distinct and stronger interaction with SARS-CoV-2 receptor ACE2 might contribute^26^. E484 mutations have been described to form a salt bridge resulting in a stronger interaction and enhanced binding to the SARS-CoV-2 receptor ACE2 has been reported^27^.

With the emergence of new variants, it is important to not only understand their virulence, infectivity and transmissibility and antigenicity but also their specific impacts on the molecular immune response. While our study helped to provide an understanding of the consequences of the E484K mutation, variants with unforeseen combinations of mutations might invoke additional unique molecular immune responses. Moving forward, immune transcriptomes from patients infected with new variants will continue to provide useful information on individual mutations or combinations of mutations. Our study also demonstrates bulk RNA-seq, with its economic very deep sequencing capacities, as an adequate tool permitting efficient and reliable identification of differently expressed genes. Analyses could be extended to identify specific antibody classes in convalescent patients and upon vaccination. Taken together, our study presents a systems view of the longitudinal and molecular immune state of hospitalized COVID-19 patients infected with SARS-CoV-2 variants carrying the E484K escape mutation throughout the disease.

As the pandemic is on-going and new variants, most recently omicron, have emerged, the information obtained from our study might inspire others to examine the pathophysiology of infection to determine if it is the same or different with each variant.

## Limitations

The findings in this report are subject to several limitations. Our study focused on hospitalized patients in a specific geographic area and on elderly patients with limited comparison to younger individuals. This study focused on the Alpha variant and the Alpha variant carrying the E484K escape mutation. The data sets from the nine patients infected with the Beta variant and the three patients infected with the Gamma variants were merely supportive. SARS-CoV-2 infections were detected and verified using PCR assays, but CT values were not recorded. No replication cohorts have been available.

## Methods

### Study population, study design and recruitment

Recruitment and blood sample collection took place between February and May 2021. This study was approved (EK Nr: 1064/2021) by the Institutional Review Board (IRB) of the Office of Research Oversight/Regulatory Affairs, Medical University of Innsbruck, Austria, which is responsible for all human research studies conducted in the State of Tyrol (Austria) regardless of whether or not the investigators have an affiliation with the University of Innsbruck. A waiver of informed consent was obtained from the Institutional Review Board (IRB) of the Office of Research Oversight/Regulatory Affairs, Medical University of Innsbruck (https://www.i-med.ac.at/ethikkommission/). This study was determined to impose minimal risk on participants. All methods were carried out in accordance with relevant guidelines and regulations. All research has been have been performed in accordance with the Declaration of Helsinki (https://www.wma.net/policies-post/wma-declaration-of-helsinki-ethical-principles-for-medical-research-involving-human-subjects/). In addition, we followed the ‘Sex and Gender Equity in Research—SAGER guidelines’ and included sex and gender considerations where relevant. Hospitalized patients infected with the Beta (B.1.351) variant have been described elsewhere^15^.

### SARS-CoV-2 virus sequencing

RNA was extracted from patient’s blood using a Maxwell RSC simply RNA Blood purification kit according to the manufacturer’s instructions (Promega, USA). Library preparation and sequencing was performed as described^28^. In short, cDNA was obtained by using reverse transcriptase with random priming. Following cDNA synthesis, primers based on sequences from the ARTICnetwork were used to generate 400 bp amplicons in two different PCR pools. After merging of pools and amplification, libraries were constructed using QIASeq FX DNA Library UDI Kit following the manufacturer’s instructions (Qiagen GmbH, North Rhine-Westphalia, Germany).

Sequencing was done with Illumina NextSeq® 500/550 using 149-bp paired-end reads with 10-bp indices (Illumina, California, USA). Obtained viral sequences were assembled using CLC Genomics Workbench v20.0.3 (Qiagen GmbH, North Rhine-Westphalia, Germany). SARS-CoV-2 isolate Wuhan-Hu-1 served as the reference genome (Accession NC_045512.2). SARS-CoV-2 variants were identified by uploading FASTA-files on freely accessible databases (http://cov-lineages.org/).

### Extraction of the buffy coat and purification of RNA

Whole blood was collected, and total RNA was extracted from the buffy coat and purified using the Maxwell RSC simply RNA Blood Kit (Promega) according to the manufacturer’s instructions. The concentration and quality of RNA were assessed by an Agilent Bioanalyzer 2100 (Agilent Technologies, CA).

### mRNA sequencing (mRNA-seq) and data analysis

The Poly-A containing mRNA was purified by poly-T oligo hybridization from 1 µg of total RNA and cDNA was synthesized using SuperScript III (Invitrogen, MA). Libraries for sequencing were prepared according to the manufacturer’s instructions with TruSeq Stranded mRNA Library Prep Kit (Illumina, CA, RS-20020595) and paired-end sequencing was done with a NovaSeq 6000 instrument (Illumina).

The raw data were subjected to QC analyses using the FastQC tool (version 0.11.9) (https://www.bioinformatics.babraham.ac.uk/projects/fastqc/). mRNA-seq read quality control was done using Trimmomatic^29^ (version 0.36) and STAR RNA-seq^30^ (version STAR 2.5.4a) using 150 bp paired-end mode was used to align the reads (hg19). HTSeq^31^ (version 0.9.1) was to retrieve the raw counts and subsequently, Bioconductor package DESeq2^32^ in R (https://www.R-project.org/) was used to normalize the counts across samples and perform differential expression gene analysis. Additionally, the RUVSeq^33^ package was applied to remove confounding factors. The data were pre-filtered keeping only genes with at least ten reads in total. The visualization was done using dplyr (https://CRAN.R-project.org/package=dplyr) and ggplot2^34^. The genes with log2 fold change > 1 or < − 1 and adjusted p-value (pAdj) < 0.05 corrected for multiple testing using the Benjamini–Hochberg method were considered significant and then conducted gene enrichment analysis (GSEA, https://www.gsea-msigdb.org/gsea/msigdb).

### Statistical analysis

Differential expression gene (DEG) identification used Bioconductor package DESeq2 in R. P-values were calculated using a paired, two-sided Wilcoxon test and adjusted p-value (pAdj) corrected using the Benjamini–Hochberg method. Genes with log2 fold change > 1 or < − 1, *p*Adj < 0.05 and > 0 value were considered significant. For significance of each GSEA category, significantly regulated gene sets were evaluated with the Kolmogorov–Smirnov statistic. Demographic data were analyzed by Chi-square on GraphPad Prism software (version 9.0.0). A value of *p < 0.05, **p < 0.001, ***p < 0.0001, ****p < 0.00001 was considered statistically significant.

### Ethics approval

This study was approved (EK Nr: 1064/2021) by the Institutional Review Board (IRB) of the Office of Research Oversight/Regulatory Affairs, Medical University of Innsbruck, Austria, which is responsible for all human research studies conducted in the State of Tyrol (Austria) regardless of whether or not the investigators have an affiliation with the University of Innsbruck.

## Supplementary Information

Supplementary Legends and Figures.

Supplementary Table 1.

Supplementary Table 2.

Supplementary Table 3.

Supplementary Table 4.

Supplementary Table 5.

Supplementary Table 6.

Supplementary Table 7.

Supplementary Table 8.

Supplementary Table 9.

Supplementary Table 10.

Supplementary Table 11.

Supplementary Table 12.

Supplementary Table 13.

## Data Availability

The RNA-seq data of COVID-19 patients infected by the Alpha or Alpha+E484K variant were uploaded under the accession GSE190680.
