# Supplementary material for: Immune transcriptome analysis of COVID-19 patients infected with SARS-CoV-2 variants carrying the E484K escape mutation identifies a distinct gene module
Source: Sci Rep. 2022 Feb 18;12:2784. doi: 10.1038/s41598-022-06752-0 (PMC8857234; doi:10.1038/s41598-022-06752-0)
Supplement: Supplementary file 1 — Supplementary Legends and Figures. [file 41598_2022_6752_MOESM1_ESM.docx]

Supplementary Information

### Immune transcriptome analysis of COVID-19 patients infected with SARS-CoV-2 variants carrying the E484K escape mutation identifies a distinct gene module

Hye Kyung Lee^1,*,†^, Ludwig Knabl^2,*,†^, Ludwig Knabl Sr.^3^, Manuel Wieser^2^, Anna Mur^4^, August Zabernigg^4^, Jana Schumacher^5^, Sebastian Kapferer^4^, Norbert Kaiser^5^,

Priscilla A. Furth^6,†^ and Lothar Hennighausen^1,†^

^1^National Institute of Diabetes, Digestive and Kidney Diseases, Bethesda, MD 20892, USA; ^2^TyrolPath, Zams, Austria; ^3^Krankenhaus St. Vinzenz, Zams, Austria; ^4^Division of Internal Medicine, Krankenhaus Kufstein, Kufstein, Austria; ^5^Division of Internal Medicine, Krankenhaus St. Johann, St. Johann, Austria; ^6^Departments of Oncology & Medicine, Georgetown University, Washington, DC, USA.

*Equal contribution

^†^Corresponding authors: HKL: [hyekyung.lee@nih.gov](mailto:hyekyung.lee@nih.gov); LK: [Ludwig.knabl@tyrolpath.at](mailto:Ludwig.knabl@tyrolpath.at); PAF: [paf3@georgetown.edu](mailto:paf3@georgetown.edu); LH: [lotharh@nih.gov](mailto:lotharh@nih.gov)

**Supplementary Tables**

**Supplementary Table 1.** Demographic and Clinical Characteristics of the study population.

**Supplementary Table 2.** List of SNPs on viral genome and amino acid changes on spike proteins. Amino acids in receptor binding domain are highlighted in yellow.

**Supplementary Table 3.** List of significantly regulated genes at non-COVID controls and hospitalized COVID-19 patients infected by Alpha variant, log2 (fold change), *p*-value and adjusted *p*-value as well as GSEA analysis.

**Supplementary Table 4.** List of significantly regulated genes at non-COVID controls and hospitalized COVID-19 patients infected by Alpha+EK variant, log2 (fold change), *p*-value and adjusted *p*-value as well as GSEA analysis.

**Supplementary Table 5.** List of significantly regulated genes at hospitalized COVID-19 patients infected by Alpha or Alpha+EK variant, log2 (fold change), *p*-value and adjusted *p*-value as well as GSEA analysis.

**Supplementary Table 6.** List of significantly regulated genes at alive and deceased COVID-19 patients infected by Alpha or Alpha+EK variant, log2 (fold change), *p*-value and adjusted *p*-value as well as GSEA analysis.

**Supplementary Table 7.** List of significantly regulated genes at hospitalized COVID-19 patients infected by multiple variants with E484K mutation, log2 (fold change), *p*-value and adjusted *p*-value as well as GSEA analysis.

**Supplementary Table 8.** List of significantly regulated genes at non-COVID controls and discharged COVID-19 patients infected by Alpha variant, log2 (fold change), *p*-value and adjusted *p*-value as well as GSEA analysis.

**Supplementary Table 9.** List of significantly regulated genes at non-COVID controls and discharged COVID-19 patients infected by Alpha+EK variant, log2 (fold change), *p*-value and adjusted *p*-value as well as GSEA analysis.

**Supplementary Table 10.** List of significantly regulated genes at non-COVID controls and convalescent COVID-19 patients infected by Alpha variant, log2 (fold change), *p*-value and adjusted *p*-value as well as GSEA analysis.

**Supplementary Table 11.** List of significantly regulated genes at non-COVID controls and convalescent COVID-19 patients infected by Alpha+EK variant, log2 (fold change), *p*-value and adjusted *p*-value as well as GSEA analysis.

**Supplementary Table 12.** List of significantly regulated genes at discharged and convalescent COVID-19 patients infected by multiple variants with E484K mutation, log2 (fold change), *p*-value and adjusted *p*-value.

**Supplementary Table 13.** List of known OAS1 SNPs that were detected in bam files of RNA-seq for each patient.

**Supplementary figures**


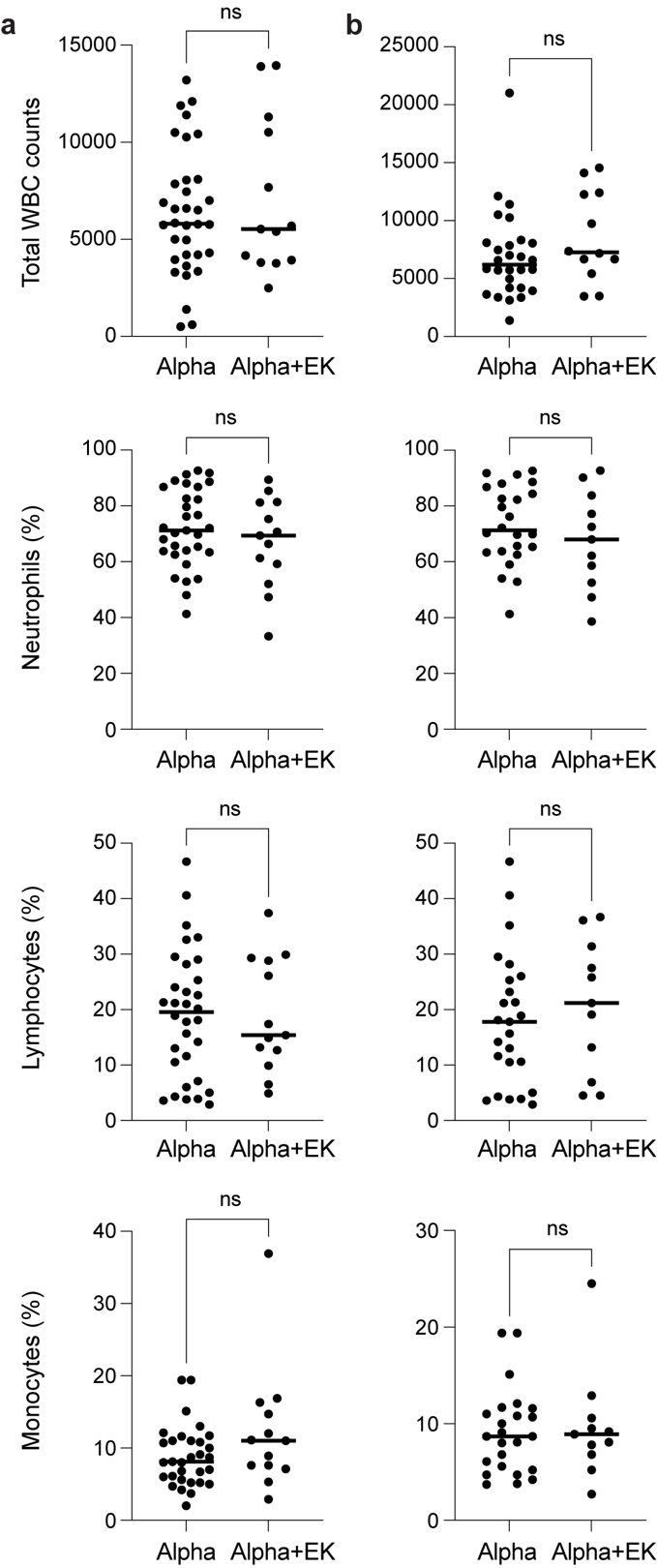


**Supplementary Figure 1. Comparison of immune cell population in PBMC of Alpha and Alpha+EK patients.** Ratios of whole blood cells, neutrophils, lymphocytes and monocytes were compared between Alpha and Alpha+EK patients in the first (a) and second (b) blood drawing.


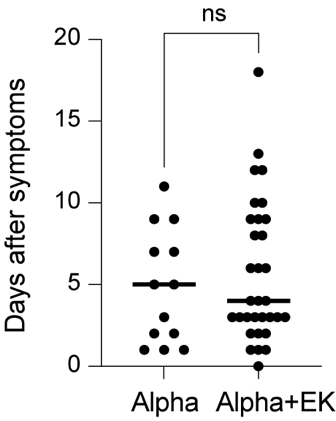


**Supplementary Figure 2.** Comparison of the first blood drawing time after symptoms in Alpha and Alpha+EK groups.
